# Author Correction: ProTInSeq: transposon insertion tracking by ultra-deep DNA sequencing to identify translated large and small ORFs

**DOI:** 10.1038/s41467-024-47153-3

**Published:** 2024-03-27

**Authors:** Samuel Miravet-Verde, Rocco Mazzolini, Carolina Segura-Morales, Alicia Broto, Maria Lluch-Senar, Luis Serrano

**Affiliations:** 1 Centre for Genomic Regulation (CRG), The Barcelona Institute of Science and Technology, Dr Aiguader 88, 08003 Barcelona, Spain; 2 Pulmobiotics, Dr Aiguader 88, 08003 Barcelona, Spain; 3 Institute of Biotechnology and Biomedicine “Vicent Villar Palasi” (IBB), Universitat Autònoma de Barcelona, Barcelona, Spain; 4 Universitat Pompeu Fabra (UPF), Barcelona, Spain; 5 ICREA, Pg. Lluis Companys 23, 08010 Barcelona, Spain; 6 Present Address: Department of Biology, Institute of Microbiology and Swiss Institute of Bioinformatics, ETH Zurich, Zurich, Switzerland

**Keywords:** Open reading frames, Next-generation sequencing, Bacterial genetics, Proteins

Correction to: *Nature Communications* 10.1038/s41467-024-46112-2, published online 7 March 2024

The original version of this Article contained an error in the Acknowledgements, which incorrectly read ‘We acknowledge the support provided by the Genomics Unit at the Centre for Genomic Regulation sequencing the samples. We also acknowledge Marc Weber for support in the analysis of Ribo-Seq data, and Veronica Raker for language editing. Funding for open access charge: ERASynBio 2nd Joint Call for Transnational Research Projects: ‘Building Synthetic Biology Capacity Through Innovative Translational Projects’, with funding from the corresponding ERASynBio National Funding Agencies; European Research Council (ERC) under the European Union’s Horizon 2020 research and innovation program [670216] (MYCOCHASSIS) by M.L.S. and L.S.; M.L.S. research has been supported by grant EUR2023-143461 “Excellence Europe” funded by AEI/10.13039/501100011033 and by the “European Union”; CERCA Programme/Generalitat de Catalunya (L.S.); and Spanish Ministry of Science and Innovation to the EMBL partnership, ‘Centro de Excelencia Severo Ochoa 2013–2017’. S.M.V. thanks Prof. Dr. Shinichi Sunagawa and the grant HFSP LT0050/2023 for support during part of the research study.’

The correct version replaces this section with ‘We acknowledge the support provided by the Genomics Unit at the Centre for Genomic Regulation sequencing the samples. We also acknowledge Marc Weber for support in the analysis of Ribo-Seq data, and Veronica Raker for language editing. This project has received funding from the European Research Council (ERC) under the European Union’s Horizon 2020 research and innovation programme MYCOCHASSIS (670216) and ERC LUNG-BIOREPAIR (101020135) ERC Advanced Grants (AdG). We also acknowledge support of the Spanish Ministry of Science and Innovation through the Centro de Excelencia Severo Ochoa (CEX2020-001049-S, MCIN/AEI /10.13039/501100011033), the Generalitat de Catalunya through the CERCA programme and to the EMBL partnership. We are grateful to the CRG Core Technologies Programme for their support and assistance in this work.’

This has been corrected in both the PDF and HTML versions of the Article.

