# Supplementary material for: ProTInSeq: transposon insertion tracking by ultra-deep DNA sequencing to identify translated large and small ORFs
Source: Nat Commun. 2024 Mar 7;15:2091. doi: 10.1038/s41467-024-46112-2 (PMC10920889; doi:10.1038/s41467-024-46112-2)
Supplement: Supplementary file 1 — Supplementary Information [file 41467_2024_46112_MOESM1_ESM.pdf]

SUPPLEMENTARY INFORMATION

SUPPLEMENTARY METHODS

Molecular cloning: Gibson assemblies of different vectors obtained in this study.

The vector TnP$_{438}$*cat*IR* was obtained after performing Gibson assembly with Cat_OI_mut, F1_Vector and F2_Vector_OR_mut fragments obtained by PCR using as template TnP$_{438}$*cat* vector and the primers described in Table S1. This vector comprises a mutation A to T in one of the fourth base of the IR sequence (IR*) that leads to the change of stop codon to Leu amino acid in the fusion protein. *Cat* gene expression is under regulation of the constitutive P$_{438}$ promoter. This vector was used to evaluate the impact of the mutation in the transposition efficacy by comparing the efficiency of transformation of with TnP$_{438}$*cat*IR* with TnP$_{438}$*cat*. The vector Tn*cat**IR* was obtained after performing Gibson assembly with Cat_OImut_Catmut, F1_Vector and F2_Vector_ORmut_Catmut fragments obtained by PCR using as template TnP$_{438}$*cat* vector and the primers described in Table S1. This vector has the IR* mutation and the P$_{438}$ and start codon have been removed from the *cat* gene (cat*). This vector was used to evaluate the translation efficacy of different genomic regions. The vector Tn*cat** was obtained after performing Gibson assembly with catMut, F1_Vector and F2_Vector_Catmut fragments obtained by PCR using as template miniTn4001Cat vector and the primers described in Table S1. The inverted repeat sequences are not mutated but it contains the cat*. This vector is the negative control to ensure that three stop codons of inverted repeat sequence ensure the abortion of translation. This vector is used to evaluate the rate of spontaneous mutants that can be resistant to chloramphenicol.

The vector TnP$_{Syn}$EryA was obtained to test if the *EryA* gene can be expressed in *M. pneumoniae*. The expression of *EryA* gene expression is under a synthetic promoter previously shown to work in different mycoplasma species (Mon. The *EryA* gene with PSyn promoter was ordered as synthetic gene (GeneScipt). Gibson assembly was performed with PSyn_EryA, F1 and F2 fragments. Those fragments were obtained by PCR using as template a TnP$_{438}$*cat* vector for F1 and F2 fragments. For the PSyn_EryA fragment the amplification was done using the ordered gene as template (Table S1). TnP$_{Syn}$EryAIR* was used to evaluate the impact of the mutation IR* in the transposition efficacy by comparing the efficiency of transformation of with TnP$_{Syn}$EryA*. The Gibson assembly was done with Irmut_PSyn_EryA, F1mut and F2_mut fragments obtained by PCR using as template the synthetic gene, TnP$_{438}$*cat* vector and the primers described in Table S1. TnEryA*IR* vector was obtained after performing Gibson assembly with Irmut_EryAMut, F1mut and F2mut fragments obtained by PCR using as template TnP$_{438}$*cat* vector or Synthetic gene (for Irmut_EryAMut fragment) and the primers described in Table S1. This vector has the IR* and the *EryA* gene without promoter neither start codon (EryA*). This vector was used to evaluate the translation efficacy of different genomic regions. The vector TnEryA* was obtained after performing Gibson assembly with EryAmut, F1 and F2 fragments obtained by PCR using as template a TnP$_{438}$*cat*

vector or synthetic gene (for EryAmut fragment) and the primers described in Table S1. The inverted repeat sequences are not mutated but it contains the EryA*. This vector is the negative control to ensure that three stop codons of inverted repeat sequence ensure the abortion of translation. This vector is used to evaluate the rate of spontaneous mutants that can be resistant to erythromycin.

The vector TnP$_{438}$barIR* was obtained by doing the Gibson assembly with A1, B1 and C1 fragments obtained by PCR using as template the synthetic gene (B1 fragment) or the TnP$_{438}$*cat*IR* vector (A1 and C1 fragments) and the primers described in Table S1. Tnbar*IR* was obtained by doing the Gibson assembly with A2, B2 and C1 fragments obtained by PCR using as template the ordered synthetic gene (B2 fragment) or the TnP$_{438}$*cat*IR* vector (A2 and C1 fragments) and the primers described in the Supplementary Table 1.

Supplementary Table 1. Details on the construction of each mini-transposon vector.

Marker	Vector	Description
Chloram phenicol	TnP$_{438}$*cat*	mini-transposon vector with the genetic cassette P$_{438}$*cat* (chloramphenicol acetyltransferase gene under the P438 constitutive promoter of the *Mycoplasma genitalium* gene *mg438 [39]*), flanked by the inverted repeat sequences (IR). It is the positive control in the transformation.
	TnP$_{438}$*cat* IR*	mini-transposon vector with the genetic cassette P$_{438}$*cat* flanked by mutated inverted repeat sequences (IR*). This vector is the positive control of the transformation with Tn*Cat**IR*. Also, it is a control to ensure that mutation in the IR does not affect the efficiency of the transposition.
	Tn*cat**IR*	mini-transposon vector with the mutated *cat* gene (*cat**, no promoter and no start codon), flanked by IR*. Viable cells after transforming with this vector should have proteins fused to cat gene in frame.
	Tn*cat**	mini-transposon vector with *cat** and IR. This is the negative control of the experiment to ensure that the selection by chloramphenicol antibiotic is correct.
Barnase	TnP$_{438}$*bar*I R*	mini-transposon vector with the genetic cassette P$_{438}$*bar* flanked by IR*. This vector is the control of the transformation with Tn*bar**IR*. All transformed cells should die. This control is similar to TnP$_{438}$*bar* but to ensure that mutation in the IR does not affect. Also the P$_{438}$*cat* cassette is cloned downstream to the *barnase* gene to select for the transformed cells.
	Tn*bar**IR*	mini-transposon vector with the mutated *barnase* gene (*bar**, no promoter and no start codon), flanked by IR*. Viable cells after transforming with this vector should not have proteins fused to the barnase gene in frame. The insertions detected with this transformation should not be found in the transformations with Tn*cat**IR* either Tn*EryA* *IR*. Also the P$_{438}$*cat* cassette is cloned downstream to the barnase gene to select for the transformed cells.
Erythro mycin	TnP$_{Syn}$*Ery A*	mini-transposon vector with the genetic cassette P$_{Syn}$*EryA* (macrolide 2'-phosphotransferase gene under the synthetic promoter [32], flanked by IR sequences. It is the positive control in the transformation.

	TnP_{Syn}*Ery* *A*IR*	mini-transposon vector with the genetic cassette P_{Syb}*EryA* flanked by IR* sequences. This vector is the positive control of the transformation with Tn*EryA**IR*. Also, it is a control to ensure that mutation in the IR does not affect the efficiency of the transposition (by comparing with TnP_{Svn}*EryA* vector).
	Tn*EryA**I R*	mini-transposon vector with the mutated *EryA* gene (*EryA**, no promoter and no start codon), flanked by IR* sequences. Viable cells after transforming with this vector should have proteins fused to EryA.
	Tn*EryA**	mini-transposon vector with *EryA**and IR sequences. This is the negative control of the experiment to ensure that the selection by erythromycin antibiotic is correct and that resistance is only acquired if cells express protein fusions.

Relating transposon analysis with transmembrane segments prediction in new and annotated ORFs

Despite not significant due to the limitations of the sizes evaluated, and the small number of new SEPs predicted to have transmembrane segments (n=39), on average in the highest selected CmB15 samples, 63 % ± 27 % of the insertions found in these smORFs were located in the TMHMM predicted cytoplasmic segments, which is lower than the results obtained for known transmembrane NE genes (81% ± 17%, one-tailed T-test P=0.12). After running the TMHMM is algorithm in 101 NE known proteins (35 lipoproteins, 66 transmembrane), our results matched the TMHMM predictions, with an error of ± 10 aa, for 41 proteins, failed in one segment in 39 (31 predicted to be cytoplasmic which are exposed in TMHMM predictions; 8 predicted to be cytoplasmic in TMHMM but found clean of insertions), and for 21 we could not do any prediction, 13 due to presenting repeated regions and the other 8 which presented at least three transmembrane segments and small non-transmembrane segments and therefore with an ir in-frame coverage was considerably reduced (21% ± 8%, one-tailed T-test P=0.12); thus preventing the efficient application of the algorithm.

SUPPLEMENTARY NOTES

We include here a full description of the Supplementary Data tables referenced in the main document:

Supplementary Data 1. Transformation efficiency

For each selection marker (column A), the library, type and replica number are associated (columns B-D, respectively). These are followed by the ratio of colonies forming units (CFU) between growing cells with antibiotic and in regular Hayflick media value, the same but in percentage scale, and normalized to respective controls (columns E-G). Columns I-O recapitulates the previous values to perform a comparison of ProTInSeq control libraries compared to a regular Tn4001 transposon, by comparing their means and standard deviation by statistical testing. Finally, columns Q to S include the mean and standard deviation values used to statistically assess the differences between ProTInSeq control and selective libraries.

Supplementary Data 2. *M. pneumoniae* base-level insertion profiles obtained with the ProTInSeq method.

For each genome base position in *M. pneumoniae* genome (column A, from 1 to 816394), we include the gene code (as *mpneu*) if corresponding to an in-frame position or 'Not-Assigned' (NA) if none and the label of the annotation (annotated for genes found in the NCBI annotation, putative for smORFs and ORFs not annotated, and non-coding if free of ORFs) for the positive (columns C and D) and negative (E and F) genome orientations. From column G to CJ, the mapping of insertions is included for every library sample sequenced in this study (0 when no insertion is found and read count (number of times insertion is found) if any). Header uses the sample identifier shared with Supplementary Data 3 by merging the reporter (Cm, Ery or Bar), library type (A/B, C/D), antibiotic concentration, strand orientation of the insertion (pos and neg, for positive and negative, respectively) and replica number.

Supplementary Data 3. ProTInSeq library description, basic statistical description, and base-level statistics by sample comparing annotated, gold-set, putative, and non-coding positions.

For each transposon library we include in columns A-G its identifier code, raw sequencing file name, reporter (Cm, Ery or Bar), library type (A/B, C/D), antibiotic concentration, strand orientation of the insertion (pos and neg, for positive and negative, respectively) and replica number. These are followed in columns H-J by the general coverage (calculated as percentage of total number of insertions retrieved over genome size, which is 816394 in *M. pneumoniae*), total reads recovered mapping and insertion and the same value in $\log_2$. Then, columns K-P include specific information only considering the fraction of the genome covering annotations associated with the specific strand orientation being explored. Following the column order, we include the size in nucleobases taken into consideration (K), number of insertions mapped (L), coverage (as the ratio between the two previous values; column M), total number of reads recovered in those positions (N), average of reads per inserted base (O), and median value of reads per inserted base (P). Columns Q-V and W-AB include this same information considering only E and NE genes, respectively, defined in the "gold set" of genes with known essentiality from the Lluch-Senar, M, *et al.* (2015) study. Finally, columns AC-AH and columns AI-AN replicate this same information

but accounting for putative annotations (every smORF and ORF not included in the NCBI annotation) and regions with no annotation associated (non-coding), respectively. These categories, except for non-coding, take into consideration only the first base every three bases as labeled in Supplementary Data 2.

Supplementary Data 4. Combined coverage and read count values by sample for each position type in *M. pneumoniae*.

For each transposon library sequenced in this study (column A), separating by the different labels annotated, putative, non-coding, and E and NE genes from the "gold" set (column B), we include the number of nucleobases considered (column C), number of insertions found in those positions (column D), coverage (as ratio between columns D and C, column E), total read count value (column F), average number of reads per insertion (column G) and standard deviation (column H). These values are used to define main figure 2A and 2C, and Extended Figure 1.

Supplementary Data 5. Paired statistical evaluation of the selection in different libraries and considering different position types in *M. pneumoniae.*

Statistical comparative by one-tailed Mann-Whitney-U between transposon libraries separated by separating by the different labels annotated, putative, non-coding, and E and NE genes from the "gold" set. Column A includes an identifier in the format library_selection_concentration_anntype1_vs_anntype2_metric. Metrics compared, in column B, can be either coverage (*cov*) or mean read count (*mean_r*). Column C includes the antibiotic concentration used to grow the cultures (notice *Barnase* library does not have a concentration assigned and *'na'* is included in those cases). Following columns include the metrics compared between two labeled base group types (1 and 2) showing the group identifiers (columns D and H); labels of the annotation types (columns E and I) that can be annotated, non-coding, putative, E and NE gold set of genes; average value (columns F and J) and standard deviations (columns G and K) used in the calculation of p-value using a one-tailed Mann-Whitney-U test (column L).

Supplementary Data 6. Available knowledge on *M. pneumoniae* M129 ORFome.

This table includes all the available information about the 30,112 sequences that could encode for a coding sequence in *M. pneumoniae*. For each identifier (column B), we include coordinates information and nucleotide and amino acid length information (columns C-H). Column I includes the gene name when the entry is found annotated in *M. pneumoniae*. Localization and function are described in columns J and K. Column L includes the operon number in which the annotation would be expressed. We also included transcription-related information average expression (column M; as $\log_2$(gene read count/gene length) and estimated average RNA copies per cell (column N) considering 4 RNA sequencing samples covering different growth times (6, 24 and 48 hours, ArrayExpress identifier E-MTAB-6203). Column O accounts for the number of mass spectrometry experiments detecting that entry (to a maximum of 116) and column P accounts for the total number of unique tryptic peptides detected. This is available for 12,426 sequences that present an amino acid length ≥19 (from 116 mass spectrometry experiments, ID PRIDE: PXD008243). Columns Q to T recapitulate protein copies per cell under different conditions (overall, extracting with urea, extracting with SDS and mean, respectively). Column U includes half-lives of the proteins.

Columns V and W describe the reference density of insertion and essentiality assigned in previous studies. Columns X and Y include the predicted RanSEPs score and ribosome binding site presence. Column Z contains information relative to homology measured against a database of smORFs from >100 bacterial species obtained in Miravet et al. 2019. This included seven groups: 0—no hits passed the thresholds defined; 1—conserved with an annotated function; 2—conserved as an annotated SEP but no associated function; 3—conserved in a different species but target and homologous sequence not found in NCBI; 4—sequence is completely or partially (> 75%) repeated ≥ 3 times in the reference genome; 5—potential pseudogene; and 6—to depict those annotations that are already found in NCBI reference annotations; column AA includes the function expected provided by this homology search. Columns AB to AD cover the output provided by Phobius, including the number of transmembrane segments, presence of signal peptide and transmembrane topology predicted by TM-HMM. Column AE includes the complex information where 1 implies that entry is functional as a monomer, 2 as dimer, and so on. Finally, columns AF-AH will be 1 if the protein is a Lon protease target, a lipoprotein, and/or a truncated gene or pseudogene, respectively, 0 otherwise.

Supplementary Data 7. ProTInSeq signal for *M. pneumoniae* M129 ORFome database.

First columns (A-AG) are shared with Supplementary Data 6. Following columns include for every sample presenting selective insertion rates in-frame using the following identifiers separated by underscores: marker (BarnB, Cm or Ery), type (control-AC or selection-BD, antibiotic concentration, sample replicate, frame measured, metric. Metrics account for number insertions in-frame (*I*), read count (*R*), linear density from non-coding regions used in the Poisson evaluation (*rNC*), probability measured (*sfNC*) and a binary for prediction (*pred*; 0 - no significant, 1 - significant). Last columns combine the number of samples each annotation has been identified. Notice for barnase library the results need to be interpreted considering it is a negative selection marker inverting the 0 and 1 meaning. This is repeated for all samples from column AH to NS. Last columns (NT-NY) combine the number of samples each annotation has been identified. Notice for barnase library the results need to be interpreted considering it is a negative selection marker inverting the 0 and 1 meaning. These same values by sample can be downloaded at Zenodo under the digital object identifier 10.5281/zenodo.7288780.

Supplementary Data 8. Summary of the identification method using gene linear densities.

For different libraries CmB, CmD and EryB, including different concentrations (columns D and E), a ROC curve study is performed retrieving the True Positive Rate (TPR; column G), False Positive Rate (FPR; column H) and Area Under the Curve (AUC; column I). The counts of ORFs estimated with this condition are expressed differentiating between *Ann* (annotated CDS in M. pneumoniae; column J), *New* (putative ORFs; column K), and *Neg* (negative control sequences; column L). The recall (percentage of candidates in each group retrieved) is also included (columns M and N). The ROC values are used to define a sample-specific threshold (*Thr*; column O), required to filter out negative control sequences and keep only those candidates that are significant with no negative control candidates. Following columns include the number of estimated proteins (also separating known SEPs in *M. pneumoniae*; column P-T). Last column includes the length in aa of the shortest ORF identified (column U). Last three rows include the mean values separating the replicas number 4 (corresponding to samples with extra passing selection, colored in purple), and the total unique ORFs identified in each category.

Supplementary Data 9. Summary for annotated proteins identified with ProTInSeq and other experimental approaches.

Summary of annotated ORFs and smORFs and the identification methods that report them. Columns A to G include the available information including gene name, start, end and strand in the genome of *M. pneumoniae*, nucleotide and amino acid length, and assigned function in NCBI. Columns H to N are used as binary classification (0 - it is not, 1 - it is in the group) for Lon target, pseudogene, split gene, lipoprotein. transmembrane, signal peptide, and annotated SEP, respectively. Column O and P represent the reference insertion density and essentiality category assigned in the Lluch-Senar, M, *et al.* (2015) study. Columns Q and R include information about how much the subsequences in the gene are repeated. Column S includes the RNA-Seq expression as $\log_2$(reads). Columns T to X include the density of insertion in-frame recovered for the selective samples from the study, summarized in column Y as detected (1) or not (0) based on the approach described. These are followed in columns Z to AB for the averaged I - insertions, R - reads, and dens - insertion density when considering those same samples. Columns AC to AH represent the ribosome footprints mapped to the genes by frame and replicate, while columns AI to AK average these same values by replicate and in total. Column AL has the Ribosome Coverage (RCV), calculated as the footprint average normalized by nucleotide length of the annotation, used to define the binary value in column AM. Finally, columns AN to AR include the information extracted from mass spectroscopy (MS), including protein copies per cell, these same values in $\log_2$ scale and the bin relative to the distribution, finishing with the protein half life (equal to -10 when no possible to compute) and a binary for detection in MS.

Supplementary Data 10. Summary of the SEPs identified with ProTInSeq, Ribo-Seq, MS and computational methods.

Summary of smORFs with significant SEP-coding potential. Columns A to G include the available information including gene name, start, end and strand in the genome of *M. pneumoniae*, nucleotide and amino acid length, and resulting translated sequence. These are followed in columns H to J for the averaged I - insertions, R - reads, and dens - insertion density when considering selective ProTInSeq samples (extracted from Supplementary Data 7). Columns K to S represent the ribosome footprints mapped to the smORFs by frame and replicate, and in average by replicate and in total. Column T has the Ribosome Coverage (RCV), calculated as the footprint average normalized by nucleotide length of the annotation, used to define the binary value in column X. Column U-AA indicates when the SEP is identified by the technique. In order, we include 1 in MS columns when the SEP is detected in one of the 106 mass spectroscopy experiments considered. Column V does the same for SEPs with significant signal by ProTInSeq. Column W has a 1 when the SEP is predicted by the computational approach RanSEPs. Column X for Ribo-Seq based on RCV values. Column Y represents SEPs validated by C_{13} in previous studies and in this study. Column Z indicates when a function has been predicted computationally for a SEP (see Supplementary Data 11). Final column AA represents when the SEP is found by BlastP against the SmProt2 list of SEPs identified by Ribo-Seq in different organisms.

Supplementary Data 11. Summary of the functions computationally predicted for SEPs.

Summary of smORFs with significant SEP-coding potential and the computationally predicted functions using different servers and bioinformatic tools. Columns A to F include the available information including gene name, start, end and strand in the genome of *M. pneumoniae*, nucleotide and amino acid length. Column G will present 0 when no information is retrieved by any of the approaches and 1 otherwise. From here, each column header is formatted as a tool:metric provided by the tool. Column H to K presents the results from AMPred, with the last column representing the total count from the three previous values when probability for antimicrobial ≥0.75. Columns L to O present the prediction of protein motifs from PfamScan. Columns P to AI has the complete output from EggNOG. DeepFRI molecular function, GO term and score are included in columns AJ to AL, while columns AM to AO present the PANNZER2 predictions for homologous search in the UniProt database. Column AP to AR present the Phobious output for signal peptide potential predicted by SignalP and transmembrane domains by TMHMM. Final columns AS and AT present the BlastP results as done in Miravet et al. 2019.

Supplementary Data 12. Estimated number of insertions to obtain similar results in other bacterial species.

Predicted number of insertions required to achieve similar identification results (highest selection conditions as in *CmB 15*, 75% of genes identified, assuming ~30% of them will be essential as observed in *M. pneumoniae*) in 108 additional bacterial genomes with diverse genome size and number of genes annotated. Column A and B represent a species code and the genome accession considered. These are followed by column C and D with the genome size in total bp and in Kbp. Column E contains the number of annotated genes in NCBI. Column F presents the total number of positions in the genome that are contained in at least one of the genes in the previous column, followed in column G by the number of expected in-frame positions (*i.e.*, first codon positions). The column H (coding ratio) is calculated as the percentage of bp in the genome associated with an annotation. Column I has the total number of ORFs with nucleotide length ≥27 bp. Columns J and K indicate the number of insertions expected with similar conditions for the total genome and for in-frame positions to retrieve comparable results to this study.

Supplementary Data 13. Ribosome footprints per genome base.

Ribosome counts per base-pair (column A) representing the number of times a ribosome is found binding an RNA in an exact genome position (discriminating by strand minus and plus) consisting of two biological replicates (RP4 and RP5) from the datasets published in ArrayExpress under the identifier E-MTAB-11935. Processed results are found in Supplementary Data 14.

Supplementary Data 14. Ribosome profiling metrics for *M. pneumoniae* ORFome.

For each locus id (column A) different metrics from the ribosome footprints are extracted. Column B and C highlight the type of annotation and alternative name when a locus identifier corresponds to an annotated gene. Columns represent the ribosome footprints mapped to the genes by frame and replicate, while columns D to L average these same values by replicate and in total. Column M has the Ribosome Coverage (RCV), calculated as the footprint average normalized by nucleotide length of the annotation evaluated.

SUPPLEMENTARY FIGURES

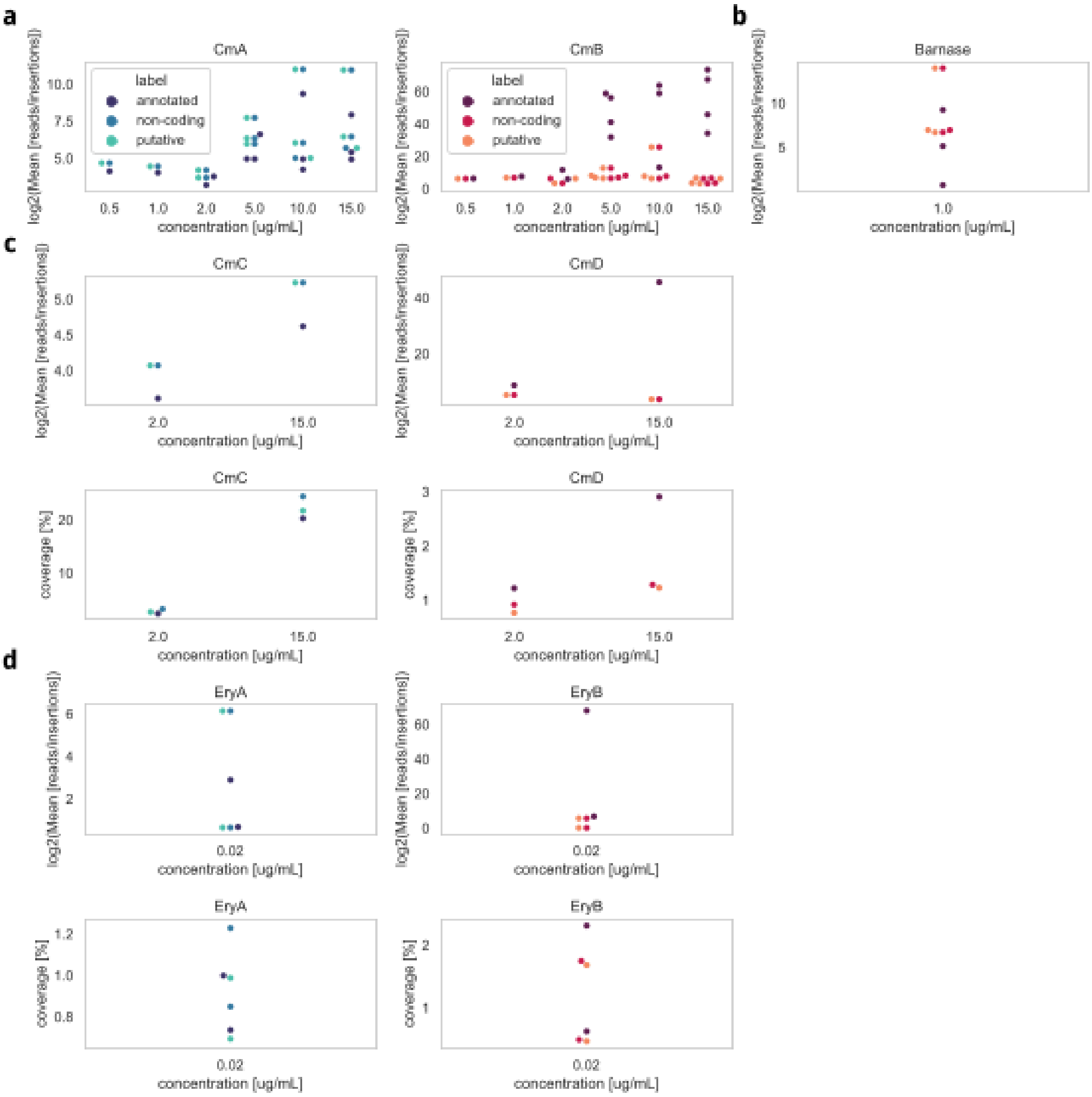

Supplementary Figure 1. Coverage and read count per insertion comparative between base annotation labels annotated, non-coding, and putative.

a, Exploration considering the $\log_2$(mean of reads/total insertions found) in the Y-axis for the *CmA* and *CmB* libraries (blues, and reds, respectively. A significant selection pattern can be observed in annotated positions after 5 µg/mL (Supplementary Data 5). Sample sizes (independent biological samples) include *CmA0.5* (n=1), *CmA1* (n=1) , *CmA2* (n=2), *CmA5* (n=3), *CmA10* (n=3) , *CmA15* (n=3), *CmB0.5* (n=1), *CmB1* (n=1) , *CmB2* (n=2), *CmB5* (n=3), *CmB10* (n=3) , *CmB15* (n=3). **b,** $\log_2$(mean of reads/total insertions) exploration for the *Barnase* libraries (n=3 independent samples). **c** and **d**, Comparative in terms of read count per insertion (top row; Y-axis) and of coverage (bottom row; Y-axis) and for the *CmC* and *CmC* libraries and *EryA* and *EryB* libraries (n=1 independent samples for each *CmC* and *CmD* concentration, and n=2 independent samples for each erythromycin concentration). In this comparisons, significant enrichment is denoted with '***' when one-tail Mann-Whitney-U test P<0.001. P-values for all the comparisons can be found in Supplementary Data 5. Source data are provided as a Source Data file.

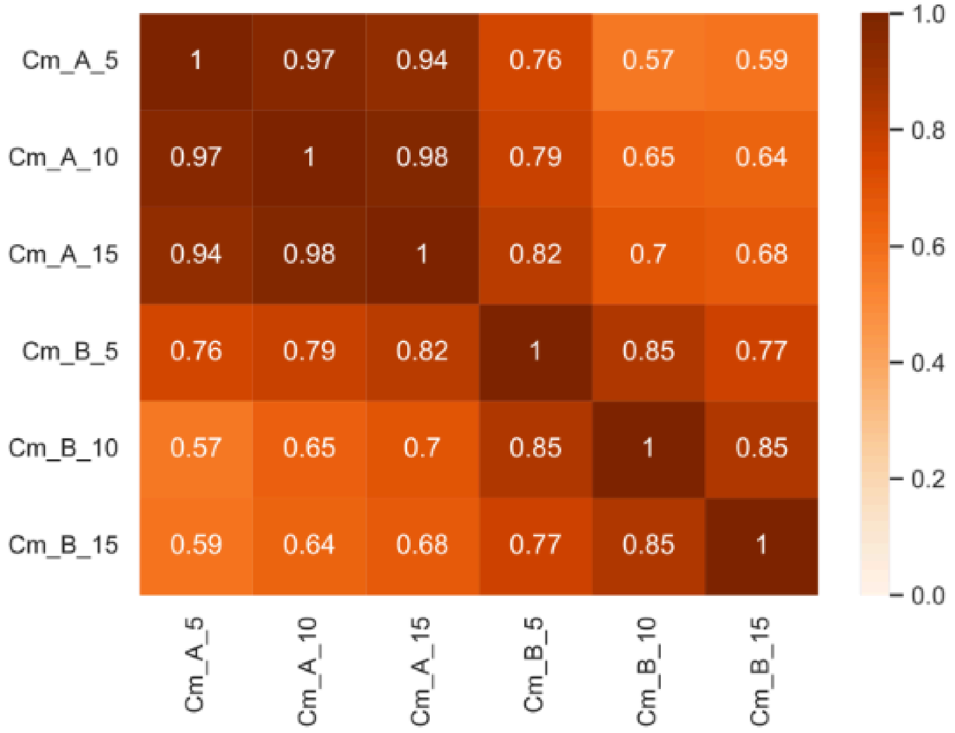

Supplementary Figure 2. Correlation in terms of gene *coverage* between selective *Cm A* and *B* libraries.

Pearson correlation (gradient) between normalized gene *coverages* obtained in the selective libraries transformed with the control and mutated chloramphenicol acetyltransferase at concentrations of 5, 10, and 15 µg/ml of chloramphenicol. It can be observed that: i) a high reproducibility is evident between concentrations within control and mutated samples, and ii) a lower correlation between control and mutated, though still significant. Source data are provided as a Source Data file.

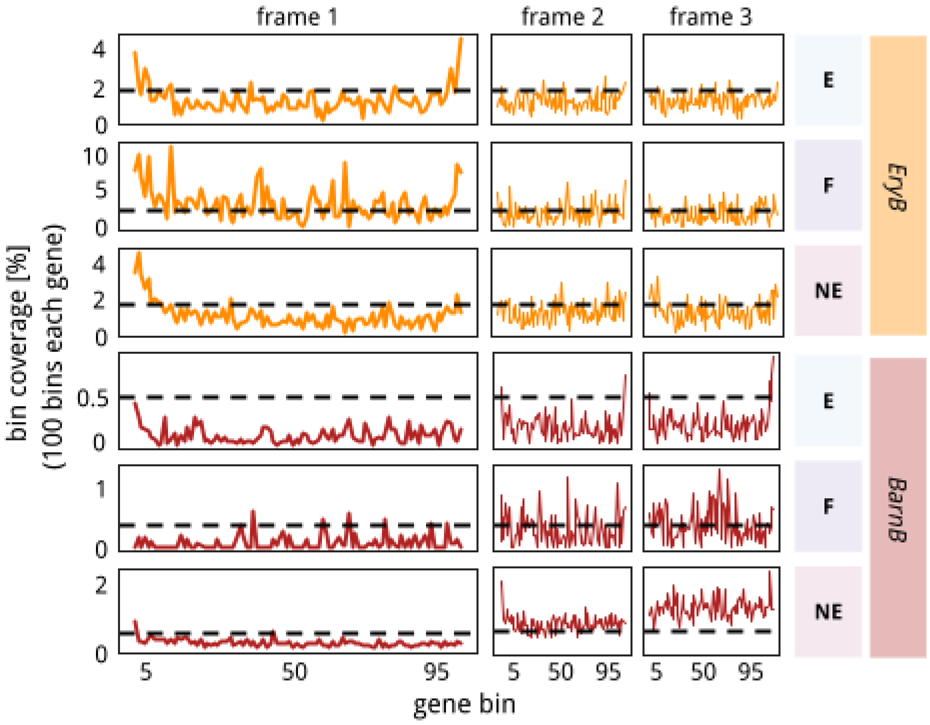

Supplementary Figure 3. Metagene comparative for *EryB* and *BarnB* libraries.

Coverage (Y-axis) calculated for genes in *M. pneumoniae*, binning them in 100 non-overlapping regions with the same size within the same gene (X-axis, from N-terminus to C-terminus). Library *EryB* (orange) presents the selection profiles observed. In this case F genes (second row) presented significant higher coverages than NE (third row; one-tail Mann-Whitney-U p-value 0.0031). Gray-dashed line is set in coverage=2%. For the *barnase* library, the specular image can be observed despite low coverage obtained (gray-dashed line at coverage= 0.5%). In this case insertions in-frame are rarely found in the population while phases 1 and 2 accumulate insertions depending on their essentiality. Source data are provided as a Source Data file.

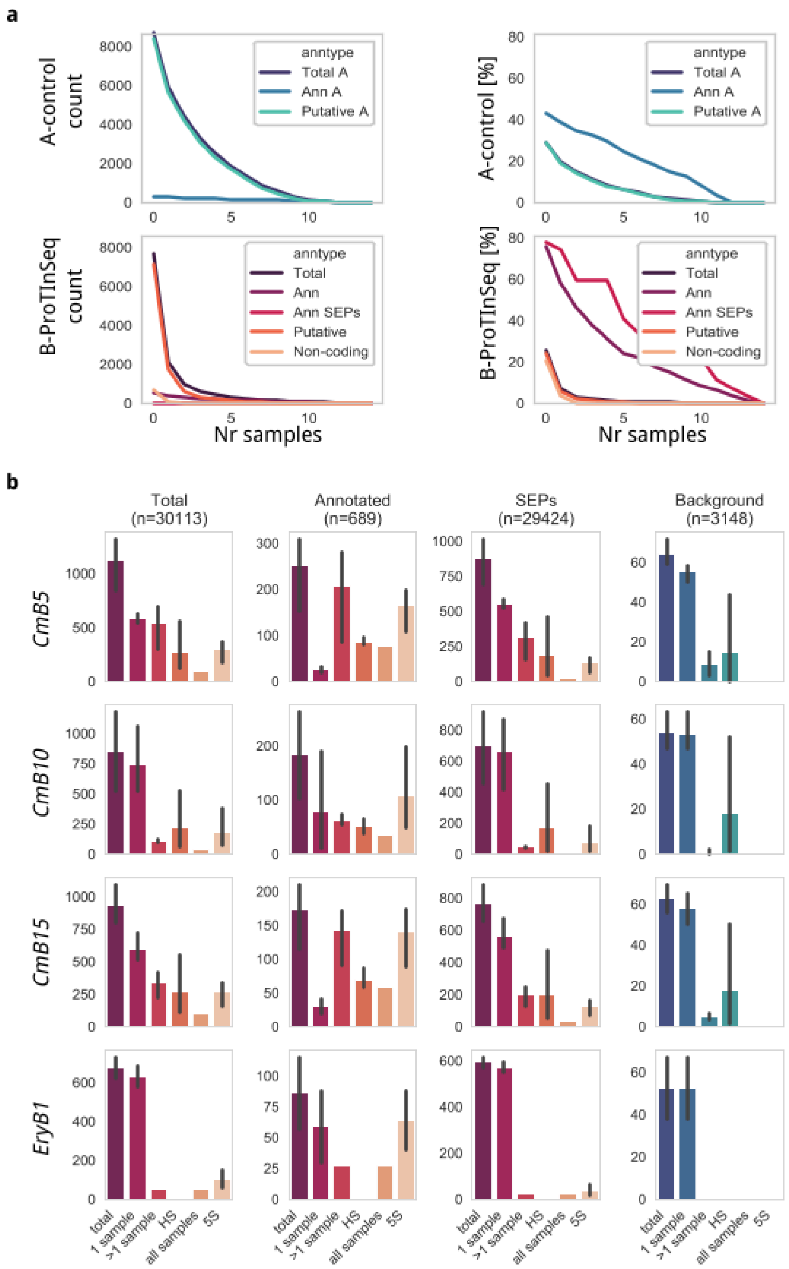

Supplementary Figure 4. Summary of the identification of insertions using ORF coverages in first codon position for *CmB* and *EryB* selective samples.

a, recall plots for the reproducibility of annotations being detected in relation to number of samples considered (X-axis) the linear density identification method for control samples (top-row) and selective ProTInSeq samples (bottom-row) representing in the Y-axis the total number of annotations retrieved (on the left) and the percentage (on the right). Line colors are associated with different annotation types including total, annotated in NCBI (*Ann*), putative ORFs and non-coding annotations. **b,** bar plots accounting in the Y-axis, by row: total number of annotations recovered, annotated genes, SEPs from putative smORFs and from the background intergenic sequences for 4 different example conditions. Error bars represent the confidence interval for the mean (bar height) between sets of samples considered in each criteria. X-axis include 6 different criteria considered: total (accumulated between all conditions), 1 samples, at least 2 samples, HS for high selection conditions (samples with replica 4 identifier in our study, corresponding to samples where additional selection passages were applied), signal in all samples and in at least 5 samples (5S). Source data are provided as a Source Data file.

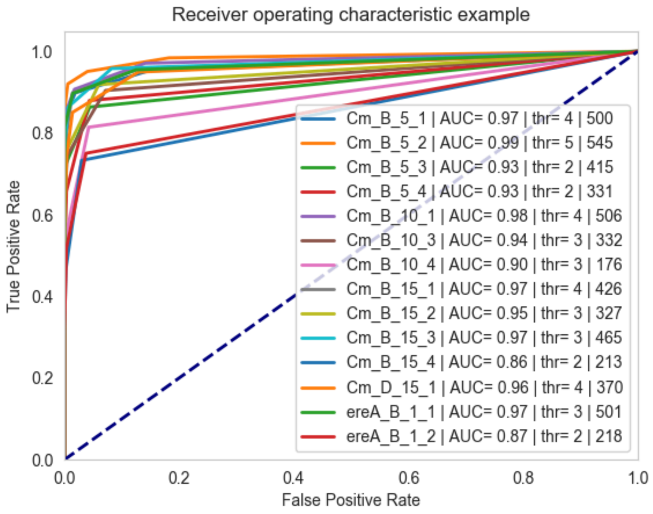

Supplementary Figure 5. ROC curves for each sample considered selected.

In the X-axis, the TPR when comparing annotated versus negative control sequences, FPR in Y-axis. The higher the TPR at lower FPR levels, the higher the AUC. Each line color represents one of the samples (separated by replicates), represented in the legend together with their AUC, the insertion threshold required to reduce to zero the FPR and the total number of proteins of *M. pneumoniae* which are recalled (n=689). Summary of the results can be found in Supplementary Data 8. Source data are provided as a Source Data file.

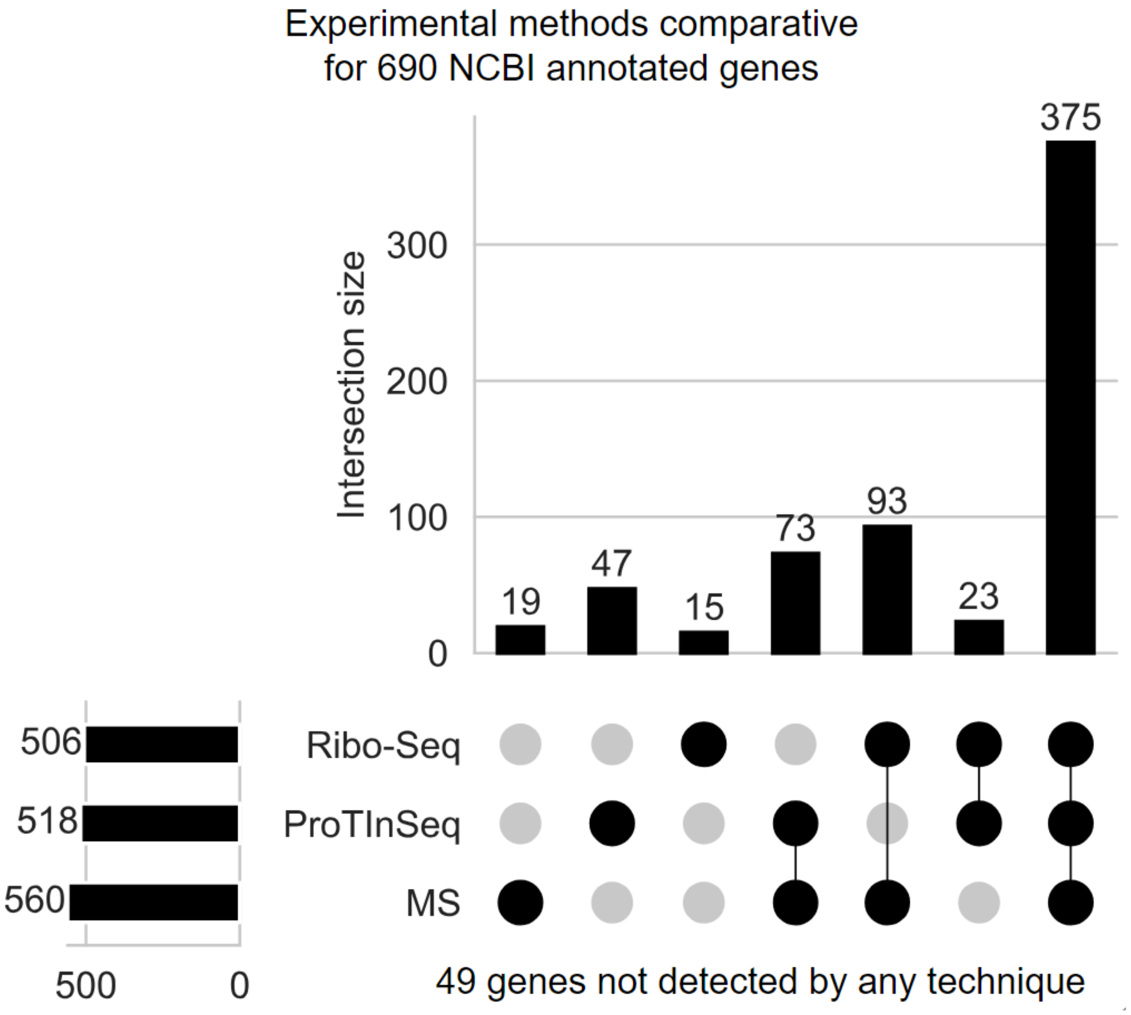

Supplementary Figure 6. Comparison of experimental methods when identifying annotated proteins.

Upset plot showing the different sets of NCBI annotated proteins in *M. pneumoniae* (n = 690, 49 not identified by any technique not included) . In the bottom plot, each row represents a method with the left barplot accounting for the number of proteins identified in each and a diagram representing intersection (black) or missing (gray). Top barplot accounts for the sizes of each method, either uniquely identified (three first columns) or intersections (rest). Source data are provided as a Source Data file.

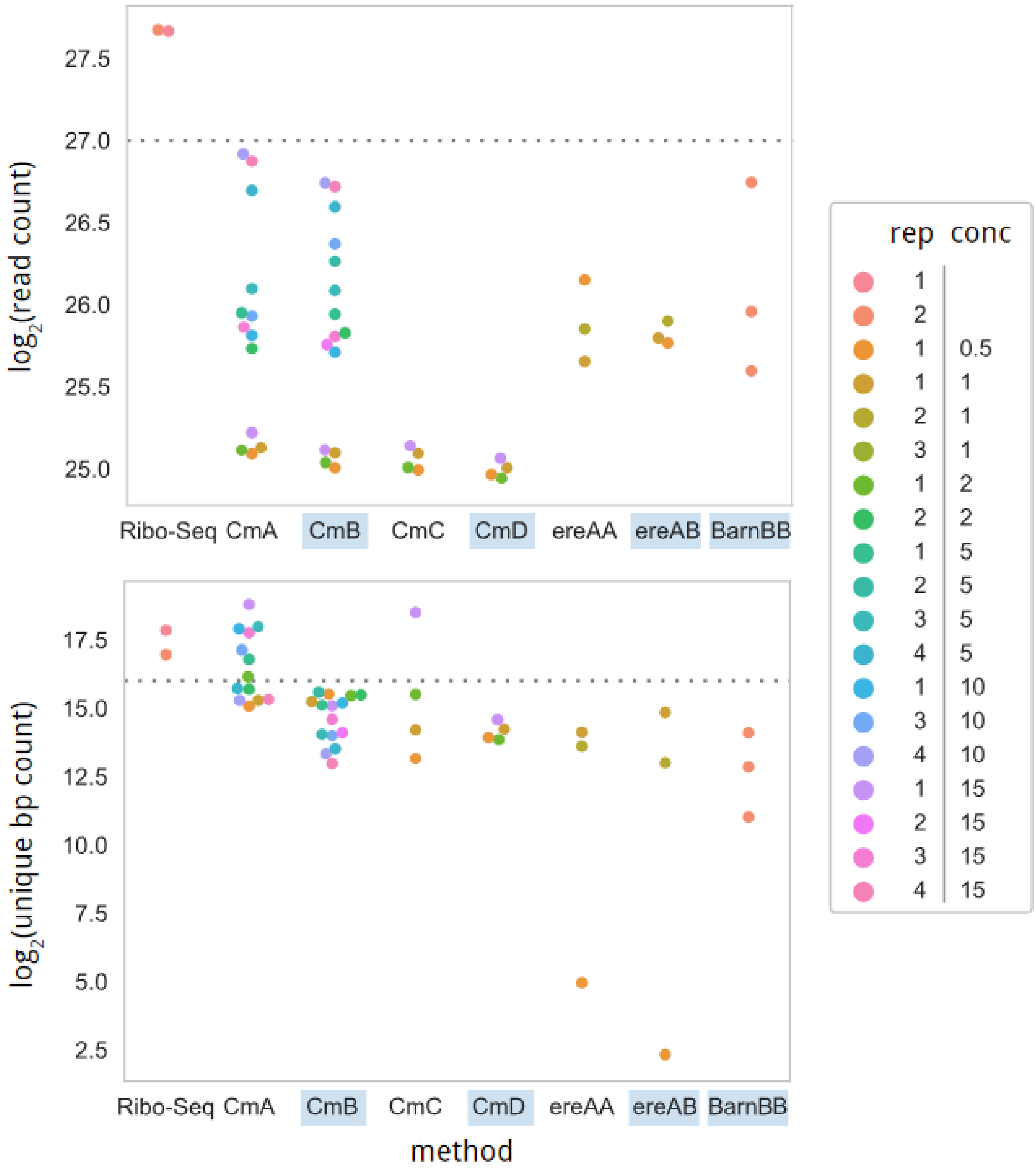

Supplementary Figure 7. Comparison in terms of read counts and unique genomic positions covered between Ribo-Seq and ProTInSeq.

Swarm plots showing the total read counts (top) and unique positions covered (bottom; representing ribosome footprints for Ribo-Seq and unique insertions for ProTInSeq) recovered for the sequencing approaches used in the study. In the X-axis it is shown first Ribo-Seq and then each of the libraries produced, showing in a blue box those samples where selection by antibiotics (Labeled as B or D for *Cm* - chloramphenicol, *Ere* - erythromycin) or by barnase (BarnB) was applied. Gray-dotted line is centered in 27 and 16 respectively for comparative purposes along the horizontal axis. Colors represent the replicate and concentration (in µg/ml units) as shown in the legend on the right. Each dot represents an independent sample (see legend for relation between replicates and antibiotic concentrations. Source data are provided as a Source Data file.

CmA	F	F	E	F	NE	F
CmB	NE	E	E	E	E	NE
RNASeq	9.17	7.04	7.96	6.75	4.06	9.18
protein cp/cell	**782.0**	ND	191.7	6.6	ND	**980.9**

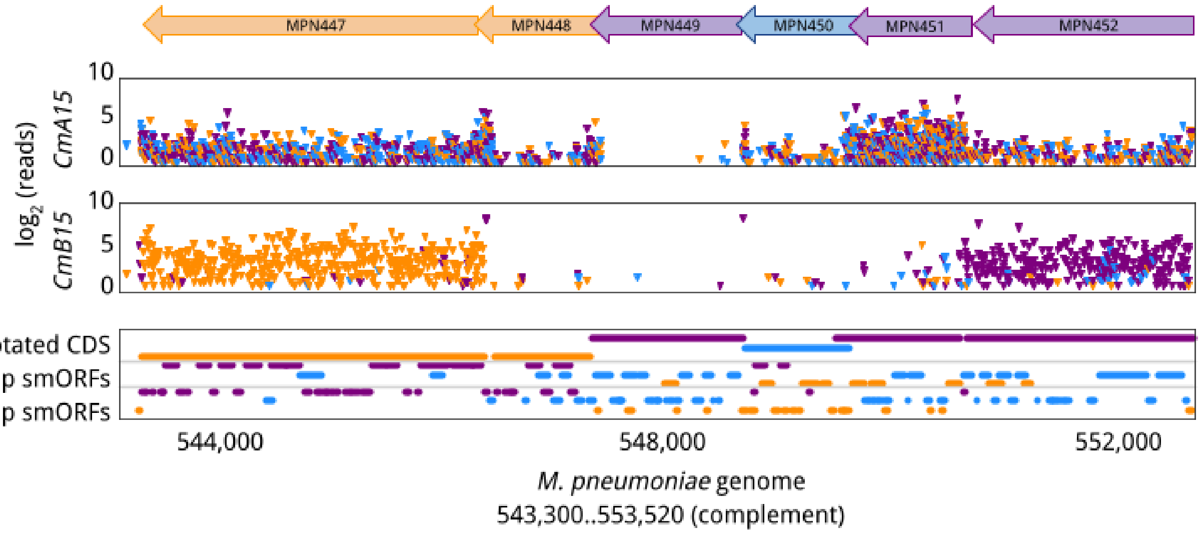

Supplementary Figure 8. Example of the relation between essentiality and protein abundance explored with ProTInSeq. From top to bottom, **Table** with the essentiality category assigned in *CmA15* and *CmB15* conditions (both replicate 1), followed by RNA-Seq measured as $\log_2$(reads/gene length) and protein abundance as copies per cell for 6 genes (arrows): *mpn447* (HMW1, attachment organelle protein), *mpn448, mpn449, mpn450* (three hypothetical proteins), *mpn451* (ComE, competence protein - like), and *mpn452* (HMW3, attachment organelle protein). **Bottom plot** shows the ORFs found, with the same frame color code as in the upper plots, distinguished by annotated proteins, smORFs between 30-300 bp, and very small ORFs (3-30 bp). We see that for NE genes in the control CmB with high protein copies per cell we find very clean profiles with in-frame insertions (orange and purple). For genes where we do not detect the protein by MS (MPN448 and MPN451) we do not see a clear preference for in-frame insertions. Finally, *mpn449*, an essential gene encoding for a conserved hypothetical protein, despite being present at 191.7 cps/cell, as it is essential only an insertion with a large number of reads is found in the N'-terminal (purple peak on the start of the gene). Source data are provided as a Source Data file.

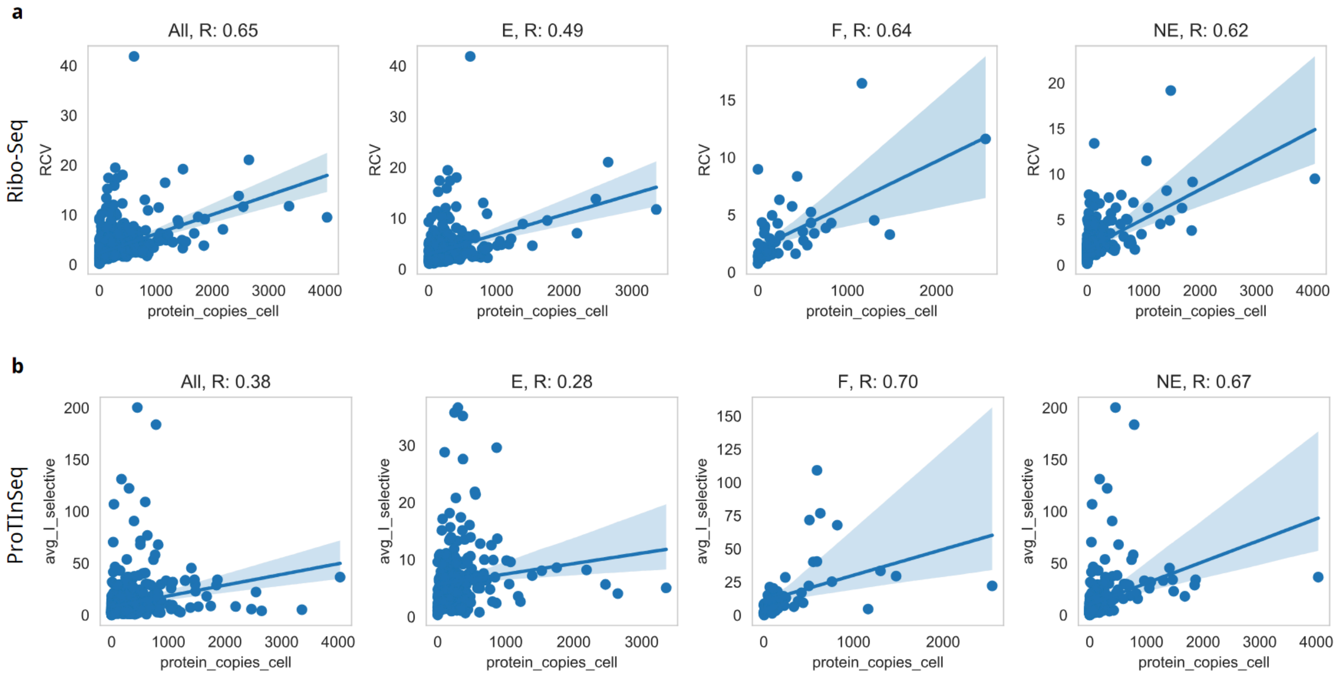

Supplementary Figure 9. Correlations between protein abundance and sequencing methods.

Regression showing the relation between protein copies per cell (X-axis) measured by mass spectroscopy for the 560 identifiable annotated proteins in *M. pneumoniae* and, in the Y-axis, Ribo-Seq RCV (top) and number of insertions recovered by ProTInSeq (bottom). Insertions were averaged between selective samples in the study (*CmB 5, 10, 15; CmD 2 and 15;* and *EryB*, n=13 independent samples). The left-most plot shows all the annotated proteins while the other shows the subsets by essentiality separating, in order, by E - essential, F - fitness, and NE - non-essential. Each dot in the graphs represents a protein while the line represents a regression with the default regplot function from seaborn package for Python. The shadow is set for a confidence interval of 95%. Source data are provided as a Source Data file.

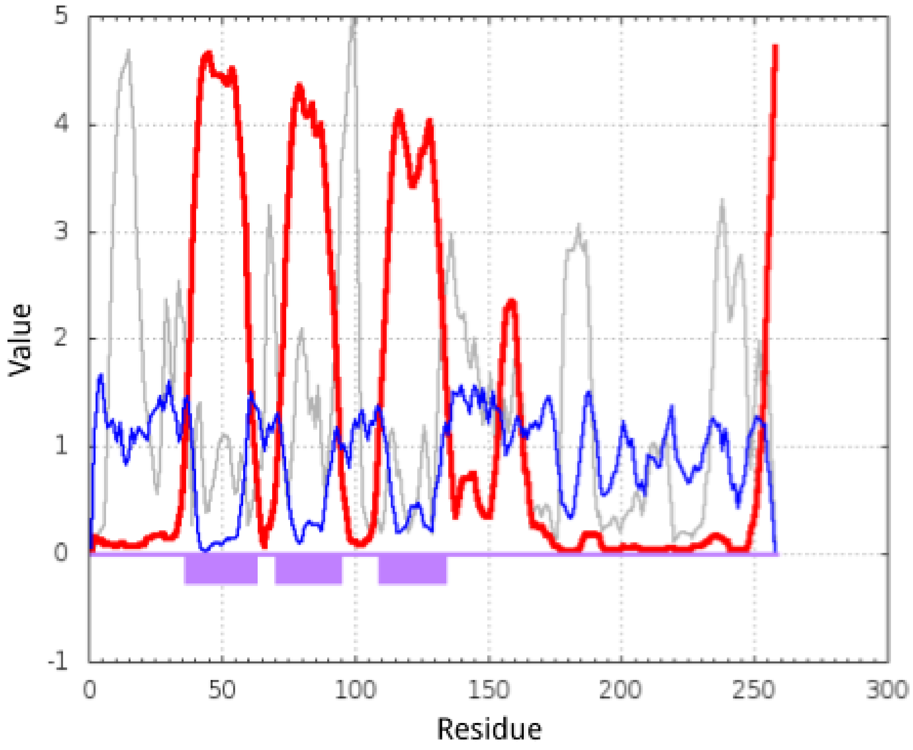

Supplementary Figure 10. Transmembrane segment prediction for *mpn359* using the SPLIT server.

Result of http://splitbioinf.pmfst.hr using the amino acid sequence (X-axis) of *mpn359* protein. Y-axis corresponds to values for i) red line: transmembrane helix preference; ii) blue line: beta preference; iii) and gray line: modified hydrophobic moment index. Violet boxes (below abscisa) correspond to the predicted transmembrane helix positions. Source data are provided as a Source Data file.

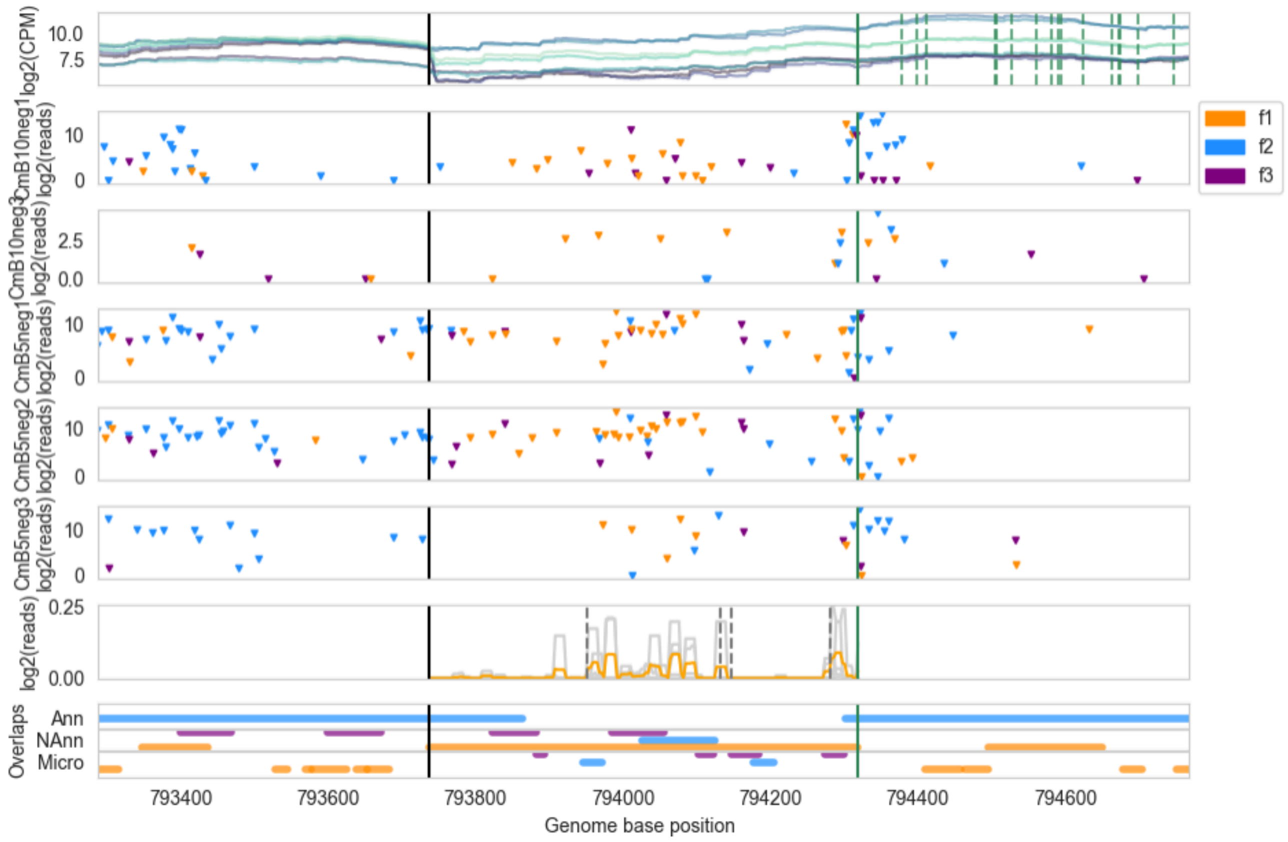

Supplementary Figure 11. Example of *mpneu25274*, a new large protein identified by ProTInSeq.
Located in the complementary strand from 794,318 to 793,737 (193 aa), *mpneu25274* is found between MPN670 and MPN671 in the two essential membrane proteins which are found in high copy numbers in *M. pneumoniae*. This protein starts with an alternative start codon. First track includes the RNA expression profile, second to sixth track shows *CmB5* and *CmB10* insertion profiles with different colors to each frame (relative to *mpneu25274*). Last two tracks include the accumulated read count value of the insertion profiles (orange line) and significant changes (gray dashed lines), and an overview of the annotations found in that genomic region. Source data are provided as a Source Data file.

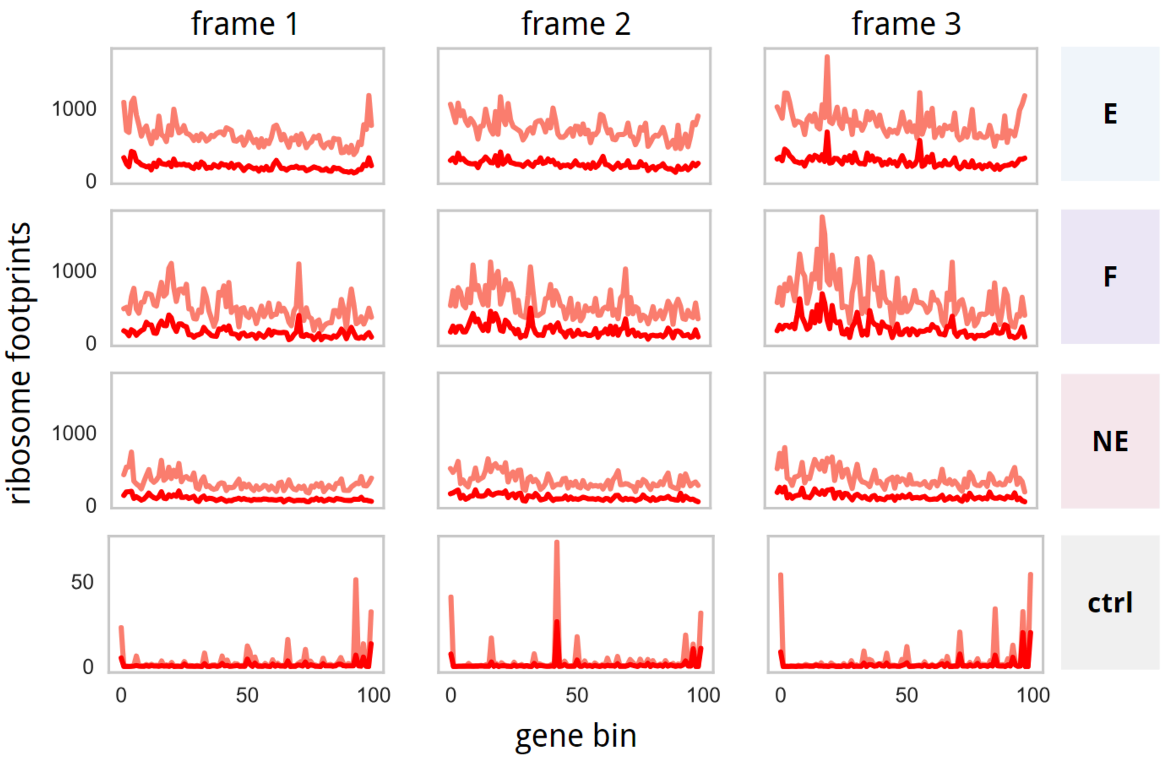

Supplementary Figure 12. Metagene profile for two ribosome profiling samples in *M. pneumoniae*.

Ribosome footprints (Y-axis) detected for genes in *M. pneumoniae*, binning them in 100 non-overlapping regions with the same size within the same gene (X-axis, from N-terminus to C-terminus). n=2 independent ribosome profiling samples are considered. Each column represents a frame of the ORF while rows represent genes by essentiality category in order: E - essential, F - fitness, and NE - non-essential. Last row is the control consisting of ncRNAs and intergenic regions. Each color is assigned to one of the experimental biological replicates. Source data are provided as a Source Data file.

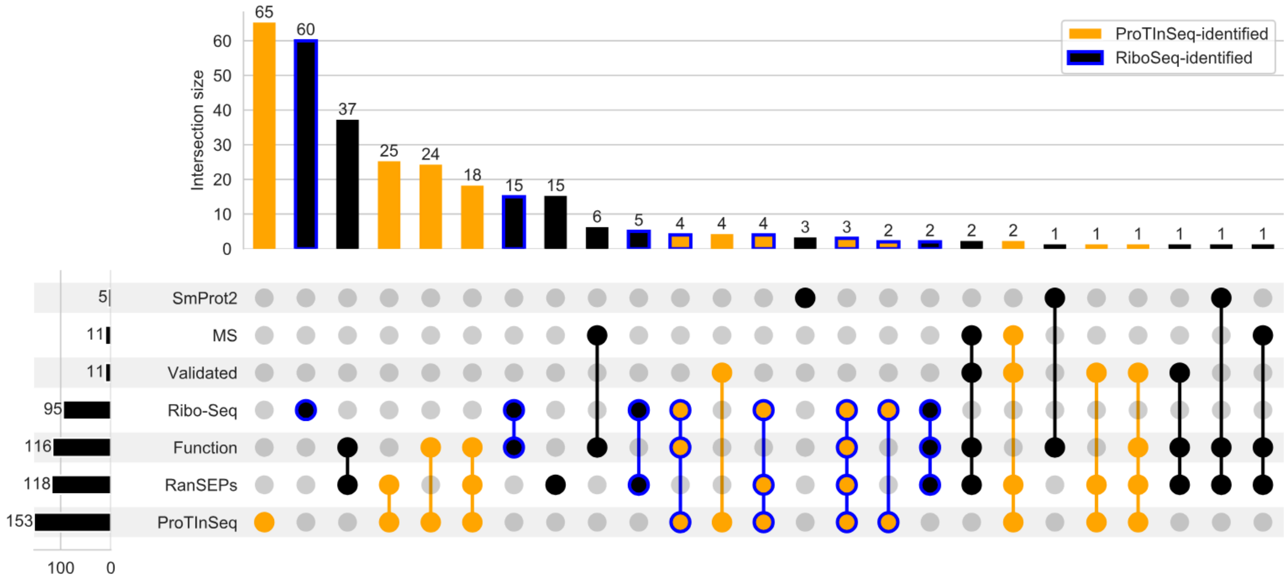

Supplementary Figure 13. Upset plot for the identification of SEPs in *M. pneumoniae* using different computational and experimental approaches.

Upset plot showing the different sets of novel SEPs found in *M. pneumoniae* with different computational (RanSEPs, Function [see upset in Extended Data Fig. 13], and SmProt2) and experimental approaches (ProTInSeq, Ribo-Seq, Validated, MS), totalling 302 SEPs. In the bottom plot, each row represents a method with the left barplot accounting for the number of proteins identified in each and a diagram representing intersection (black) or missing (gray). Top barplot accounts for the sizes of each method, either uniquely identified (three first columns) or intersections (rest). Color orange appears for those cases where ProTInSeq identifies proteins in that set. Blue lines do the same but for Ribo-Seq identified SEPs. Source data are provided as a Source Data file.

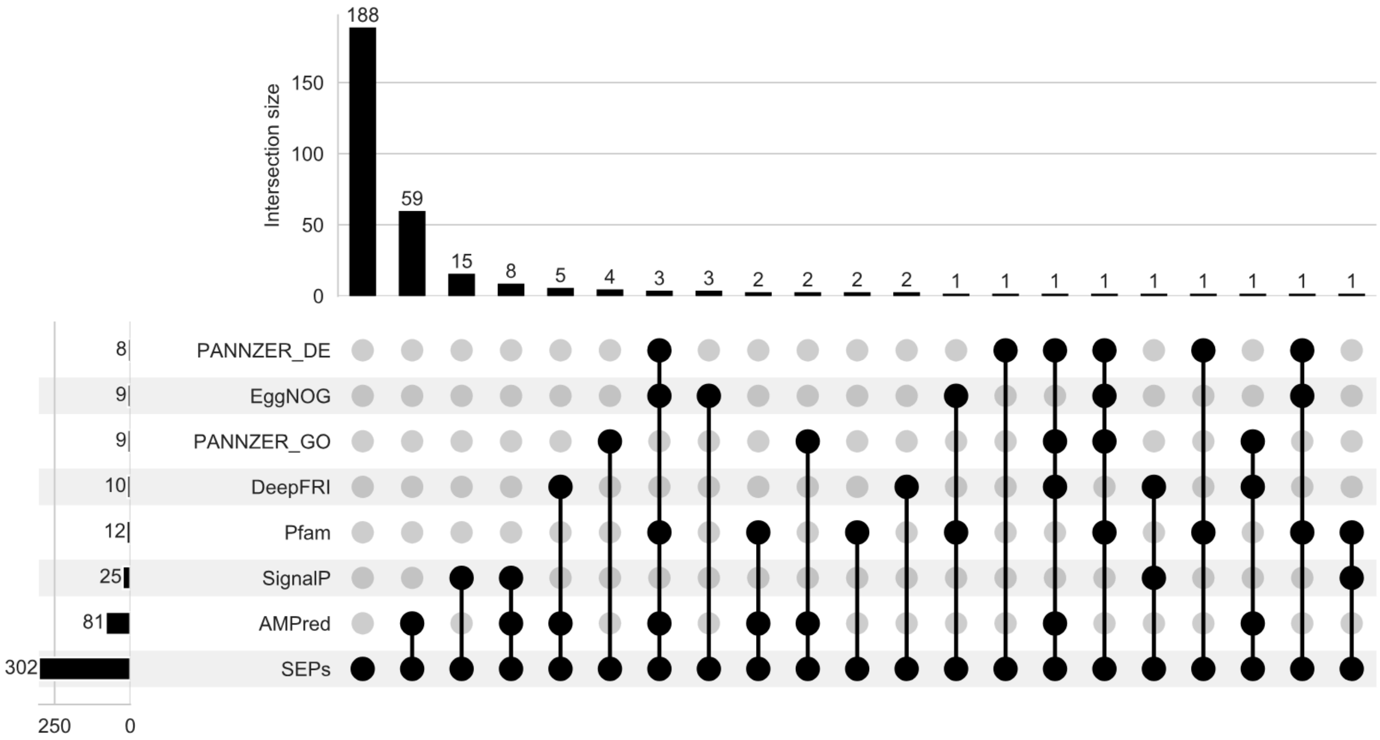

Supplementary Figure 14. Upset plot showing the landscape of function prediction for SEPs using different computational approaches.

Upset plot showing the function prediction by different computational approaches for 302 SEPs identified in *M. pneumoniae*. In the bottom plot, each row represents a method with the left barplot accounting for the number of proteins identified in each and a diagram representing intersection (black) or missing (gray). Top barplot accounts for the sizes of each method, either uniquely identified (three first columns) or intersections (rest). Notice AMPred, which identifies antimicrobial proteins, is the second larger set. Source data are provided as a Source Data file.

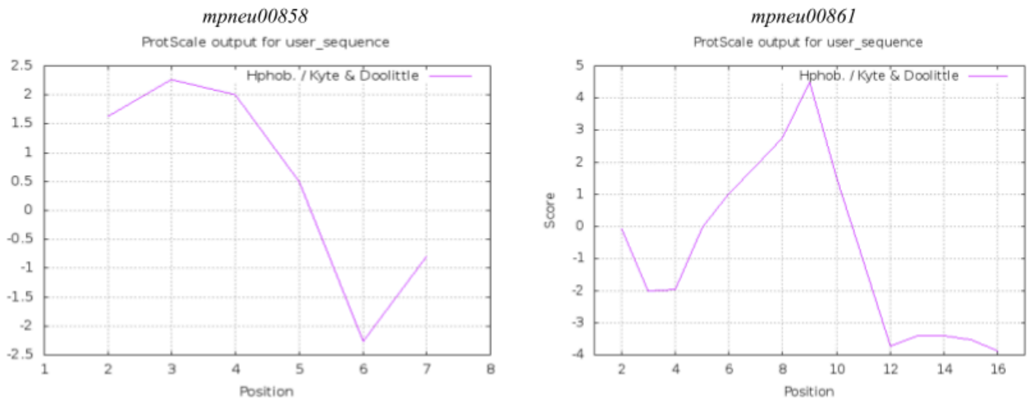

Supplementary Figure 15. Hydrophobicity profile measured for *mpneu00858* and *mpneu00861*

Hydrophobicity profile by amino acid position measured with Kyte J., Doolittle R.F. method (J. Mol. Biol. 157:105-132(1982)) using the ProtScale tool website https://web.expasy.org/protscale/. The hydrophobicity (Y-axis) peaks can be observed at amino acid positions (X-axis) 3 and 9, respectively. Source data are provided as a Source Data file.
