# Supplementary material for: ProTInSeq: transposon insertion tracking by ultra-deep DNA sequencing to identify translated large and small ORFs
Source: Nat Commun. 2024 Mar 7;15:2091. doi: 10.1038/s41467-024-46112-2 (PMC10920889; doi:10.1038/s41467-024-46112-2)
Supplement: Supplementary file 2 — Reporting Summary [file 41467_2024_46112_MOESM2_ESM.pdf]

nature portfolio

Corresponding author(s): Samuel Miravet-Verde, Luis Serrano

Last updated by author(s): Feb 8, 2024

Reporting Summary

Nature Portfolio wishes to improve the reproducibility of the work that we publish. This form provides structure for consistency and transparency in reporting. For further information on Nature Portfolio policies, see our Editorial Policies and the Editorial Policy Checklist.

Statistics

For all statistical analyses, confirm that the following items are present in the figure legend, table legend, main text, or Methods section.

n/a	Confirmed	
☐	☒	The exact sample size (*n*) for each experimental group/condition, given as a discrete number and unit of measurement
☐	☒	A statement on whether measurements were taken from distinct samples or whether the same sample was measured repeatedly
☐	☒	The statistical test(s) used AND whether they are one- or two-sided<br>*Only common tests should be described solely by name; describe more complex techniques in the Methods section.*
☐	☒	A description of all covariates tested
☐	☒	A description of any assumptions or corrections, such as tests of normality and adjustment for multiple comparisons
☐	☒	A full description of the statistical parameters including central tendency (e.g. means) or other basic estimates (e.g. regression coefficient) AND variation (e.g. standard deviation) or associated estimates of uncertainty (e.g. confidence intervals)
☐	☒	For null hypothesis testing, the test statistic (e.g. *F*, *t*, *r*) with confidence intervals, effect sizes, degrees of freedom and *P* value noted<br>*Give P values as exact values whenever suitable.*
☒	☐	For Bayesian analysis, information on the choice of priors and Markov chain Monte Carlo settings
☒	☐	For hierarchical and complex designs, identification of the appropriate level for tests and full reporting of outcomes
☐	☒	Estimates of effect sizes (e.g. Cohen's *d*, Pearson's *r*), indicating how they were calculated

Our web collection on statistics for biologists contains articles on many of the points above.

Software and code

Policy information about availability of computer code

Data collection

Software HiSeq Control Software 2.2.58

Data analysis

The Tn-Seq processing tool FastQins 1.0 (https://github.com/CRG-CNAG/fastqins) was used to analyse the sequencing results presented in this work. This tool has the following dependencies:
- FastUniq 1.1
- Bowtie 2 2.5.2
- Samtools 1.18
- Bedtools 2.31
- ruffus 2.8.4 (Python pipeline management)

Python libraries used for statistical tests and plotting:
- protinseq 1.0 (https://github.com/samuelmiver/protinseq) DOI: 10.5281/zenodo.10637277
- anubis 1.0 (https://github.com/CRG-CNAG/anubis)
- scikit-learn 1.3
- matplotlib 3.8
- seaborn 0.13.0
- scipy 1.11.3

Python libraries used for identification of transmembrane segments:
- ruptures 1.1.9

- tmhmm 1.0

For manuscripts utilizing custom algorithms or software that are central to the research but not yet described in published literature, software must be made available to editors and reviewers. We strongly encourage code deposition in a community repository (e.g. GitHub). See the Nature Portfolio guidelines for submitting code & software for further information.

Data

Policy information about availability of data

All manuscripts must include a data availability statement. This statement should provide the following information, where applicable:

- Accession codes, unique identifiers, or web links for publicly available datasets
- A description of any restrictions on data availability
- For clinical datasets or third party data, please ensure that the statement adheres to our policy

The transposon sequencing raw data generated in this study have been deposited in the ArrayExpress database under accession code E-MTAB-10380, available in the following the link https://www.ebi.ac.uk/biostudies/arrayexpress/studies/E-MTAB-10380.

The RNA-Seq raw sequencing files used in this study can be accessed in ArrayExpress under the identifier E-MTAB-6203 or accessing the project link https://www.ebi.ac.uk/arrayexpress/experiments/E-MTAB-6203.

The ribosome profiling sequencing data for M. pneumoniae used in this study can be accessed in ArrayExpress under the identifier E-MTAB-11935, accessible using the link https://www.ebi.ac.uk/biostudies/arrayexpress/studies/E-MTAB-11935.

The mass spectroscopy datasets for M. pneumoniae used in this study can be accessed in the PRIDE database under the identifier PXD008243 accessible using the link https://www.ebi.ac.uk/pride/archive/projects/PXD008243.

The processed ProTInSeq data explored in this work, including base- and gene-level insertion mappings, can be found at Zenodo with the record ID 7288780 and Digital Object Identifier 10.5281/zenodo.7288779 accessible using the link https://zenodo.org/records/7288780.

Research involving human participants, their data, or biological material

Policy information about studies with human participants or human data. See also policy information about sex, gender (identity/presentation), and sexual orientation and race, ethnicity and racism.

Reporting on sex and gender	NA
Reporting on race, ethnicity, or other socially relevant groupings	NA
Population characteristics	NA
Recruitment	NA
Ethics oversight	NA

Note that full information on the approval of the study protocol must also be provided in the manuscript.

Field-specific reporting

Please select the one below that is the best fit for your research. If you are not sure, read the appropriate sections before making your selection.

- [x] Life sciences
- [ ] Behavioural & social sciences
- [ ] Ecological, evolutionary & environmental sciences

For a reference copy of the document with all sections, see nature.com/documents/nr-reporting-summary-flat.pdf

Life sciences study design

All studies must disclose on these points even when the disclosure is negative.

Sample size	A minimum sample size of n=2 was ensured for every sample used in the study. For samples used in downstream relevant analysis, n=3 and n=4 was considered.
Data exclusions	No data excluded.
Replication	The number of independent replication experiments (36) by library tested included:<br>- Cm_A_0.5 n=1<br>- Cm_A_1 n=1<br>- Cm_A_2 n=1<br>- Cm_A_5 n=3<br>- Cm_A_10 n=3

- Cm_A_15 n=3
- Cm_B_0.5 n=1
- Cm_B_1 n=1
- Cm_B_2 n=2
- Cm_B_5 n=4
- Cm_B_10 n=3
- Cm_B_15 n=4
- Cm_C_2 n=1
- Cm_C_15 n=1
- Cm_D_2 n=1
- Cm_D_15 n=1
- ereA_A_0.02 n=2
- ereA_B_0.02 n=2
- BarnB_B_1 n=3

Randomization

Samples were allocated into different groups based on the experiment conducted:

- Samples Cm_A, Cm_C, and ereA_A were utilized independently as controls. Libraries transformed with the same construct and cultivated under identical antibiotic concentrations were merged (either by averaging or summing up) after confirming a high correlation among replicates.
- ProTInSeq libraries examined in samples Cm_B, Cm_D, and ereA_D served as controls, combined by library strategy.
- For most analyses, control replicates were juxtaposed with ProTInSeq samples, each combined by library and concentration.
- For identification purposes, ProTInSeq samples exhibiting significant in-frame selection were amalgamated (Cm_B_5, Cm_B_10, Cm_B_15, Cm_D_2, Cm_D_15, ereA_B_0.02).
- Barnase samples (BarnB) were independently analyzed. No antibiotic concentrations were tested with the negative-selection marker as they were not applicable.

The main document includes the specific comparisons conducted in each case. Before merging sample groups, we ensured reproducibility by correlating the number of insertions per gene and in the whole genome across libraries.

Blinding

Samples were processed and analysed in the exact same manner, thus, no blinding was required. Type of sample was used in final steps to describe and represent the particular results.

Reporting for specific materials, systems and methods

We require information from authors about some types of materials, experimental systems and methods used in many studies. Here, indicate whether each material, system or method listed is relevant to your study. If you are not sure if a list item applies to your research, read the appropriate section before selecting a response.

Materials & experimental systems

n/a	Involved in the study
☒	☐ Antibodies
☒	☐ Eukaryotic cell lines
☒	☐ Palaeontology and archaeology
☒	☐ Animals and other organisms
☒	☐ Clinical data
☒	☐ Dual use research of concern
☒	☐ Plants

Methods

n/a	Involved in the study
☒	☐ ChIP-seq
☒	☐ Flow cytometry
☒	☐ MRI-based neuroimaging

Plants

Seed stocks

Report on the source of all seed stocks or other plant material used. If applicable, state the seed stock centre and catalogue number. If plant specimens were collected from the field, describe the collection location, date and sampling procedures.

Novel plant genotypes

Describe the methods by which all novel plant genotypes were produced. This includes those generated by transgenic approaches, gene editing, chemical/radiation-based mutagenesis and hybridization. For transgenic lines, describe the transformation method, the number of independent lines analyzed and the generation upon which experiments were performed. For gene-edited lines, describe the editor used, the endogenous sequence targeted for editing, the targeting guide RNA sequence (if applicable) and how the editor was applied.

Authentication

Describe any authentication procedures for each seed stock used or novel genotype generated. Describe any experiments used to assess the effect of a mutation and, where applicable, how potential secondary effects (e.g. second site T-DNA insertions, mosiacism, off-target gene editing) were examined.
