# Supplementary material for: ProTInSeq: transposon insertion tracking by ultra-deep DNA sequencing to identify translated large and small ORFs
Source: Nat Commun. 2024 Mar 7;15:2091. doi: 10.1038/s41467-024-46112-2 (PMC10920889; doi:10.1038/s41467-024-46112-2)
Supplement: Supplementary file 3 — Description of Additional Supplementary Files [file 41467_2024_46112_MOESM3_ESM.pdf]

Title: Supplementary Data 1

Description: **Transformation efficiency**
For each selection marker (column A), the library, type and replica number are associated (columns B-D, respectively). These are followed by the ratio of colonies forming units (CFU) between growing cells with antibiotic and in regular Hayflick media value, the same but in percentage scale, and normalized to respective controls (columns E-G). Columns I-O recapitulates the previous values to perform a comparison of ProTInSeq control libraries compared to a regular Tn4001 transposon, by comparing their means and standard deviation by statistical testing. Finally, columns Q to S include the mean and standard deviation values used to statistically assess the differences between ProTInSeq control and selective libraries.

Title: Supplementary Data 2

Description: ***M. pneumoniae* base-level insertion profiles obtained with the ProTInSeq method.**
For each genome base position in *M. pneumoniae* genome (column A, from 1 to 816394), we include the gene code (as *mpneu*) if corresponding to an in-frame position or 'Not-Assigned' (NA) if none and the label of the annotation (annotated for genes found in the NCBI annotation, putative for smORFs and ORFs not annotated, and non-coding if free of ORFs) for the positive (columns C and D) and negative (E and F) genome orientations. From column G to CJ, the mapping of insertions is included for every library sample sequenced in this study (0 when no insertion is found and read count (number of times insertion is found) if any). Header uses the sample identifier shared with Supplementary Data 3 by merging the reporter (Cm, Ery or Bar), library type (A/B, C/D), antibiotic concentration, strand orientation of the insertion (pos and neg, for positive and negative, respectively) and replica number.

Title: Supplementary Data 3

Description: **ProTInSeq library description, basic statistical description, and base-level statistics by sample comparing annotated, gold-set, putative, and non-coding positions.**
For each transposon library we include in columns A-G its identifier code, raw sequencing file name, reporter (Cm, Ery or Bar), library type (A/B, C/D), antibiotic concentration, strand orientation of the insertion (pos and neg, for positive and negative, respectively) and replica number. These are followed in columns H-J by the general coverage (calculated as percentage of total number of insertions retrieved over genome size, which is 816394 in *M. pneumoniae*), total reads recovered mapping and insertion and the same value in $log_2$. Then, columns K-P include specific information only considering the fraction of the genome covering annotations associated with the specific strand orientation being explored. Following the column order, we include the size in nucleobases taken into consideration (K), number of insertions mapped (L), coverage (as the ratio between the two previous values; column M), total number of reads recovered in those positions (N), average of reads per inserted base (O), and median value of reads per inserted base (P). Columns Q-V and W-AB include this same information considering only E and NE genes, respectively, defined in the "gold set" of genes with known essentiality from the Lluch-Senar, M, *et al.* (2015) study. Finally, columns AC-AH and columns AI-AN replicate this same information but accounting for putative annotations (every smORF and ORF not included in the NCBI annotation) and regions with no annotation associated (non-coding), respectively. These categories, except for non-coding, take into consideration only the first base every three bases as labeled in Supplementary Data 2.

Title: Supplementary Data 4

Description: **Combined coverage and read count values by sample for each position type in *M. pneumoniae*.**
For each transposon library sequenced in this study (column A), separating by the different labels annotated, putative, non-coding, and E and NE genes from the "gold" set (column B), we include the number of nucleobases considered (column C), number of insertions found in those positions (column D), coverage (as ratio between columns D and C, column E), total read count value (column F), average number of reads per insertion (column G) and standard deviation (column H). These values are used to define main figure 2A and 2C, and Extended Figure 1.

Title: Supplementary Data 5

Description: **Paired statistical evaluation of the selection in different libraries and considering different position types in *M. pneumoniae.***
Statistical comparative by one-tailed Mann-Whitney-U between transposon libraries separated by separating by the different labels annotated, putative, non-coding, and E and NE genes from the "gold" set. Column A includes an identifier in the format library_selection_concentration_anntype1_vs_anntype2_metric. Metrics compared, in column B, can be either coverage (*cov*) or mean read count (*mean_r*). Column C includes the antibiotic concentration used to grow the cultures (notice *Barnase* library does not have a concentration assigned and *'na'* is included in those cases). Following columns include the metrics compared between two labeled base group types (1 and 2) showing the group identifiers (columns D and H); labels of the annotation types (columns E and I) that can be annotated, non-coding, putative, E and NE gold set of genes; average value (columns F and J) and standard deviations (columns G and K) used in the calculation of p-value using a one-tailed Mann-Whitney-U test (column L).

Title: Supplementary Data 6
Description: **Available knowledge on *M. pneumoniae* M129 ORFome.**
This table includes all the available information about the 30,112 sequences that could encode for a coding sequence in *M. pneumoniae*. For each identifier (column B), we include coordinates information and nucleotide and amino acid length information (columns C-H). Column I includes the gene name when the entry is found annotated in *M. pneumoniae*. Localization and function are described in columns J and K. Column L includes the operon number in which the annotation would be expressed. We also included transcription-related information average expression (column M; as $\log_2$(gene read count/gene length) and estimated average RNA copies per cell (column N) considering 4 RNA sequencing samples covering different growth times (6, 24 and 48 hours, ArrayExpress identifier E-MTAB-6203). Column O accounts for the number of mass spectrometry experiments detecting that entry (to a maximum of 116) and column P accounts for the total number of unique tryptic peptides detected. This is available for 12,426 sequences that present an amino acid length ≥19 (from 116 mass spectrometry experiments, ID PRIDE: PXD008243). Columns Q to T recapitulate protein copies per cell under different conditions (overall, extracting with urea, extracting with SDS and mean, respectively). Column U includes half-lives of the proteins. Columns V and W describe the reference density of insertion and essentiality assigned in previous studies. Columns X and Y include the predicted RanSEPs score and ribosome binding site presence. Column Z contains information relative to homology measured against a database of smORFs from >100 bacterial species obtained in Miravet et al. 2019. This included seven groups: 0—no hits passed the thresholds defined; 1—conserved with an annotated function; 2—conserved as an annotated SEP but no associated function; 3—conserved in a different species but target and homologous sequence not found in NCBI; 4—sequence is completely or partially (> 75%) repeated ≥ 3 times in the reference genome; 5—potential pseudogene; and 6—to depict those annotations that are already found in NCBI

reference annotations; column AA includes the function expected provided by this homology search. Columns AB to AD cover the output provided by Phobius, including the number of transmembrane segments, presence of signal peptide and transmembrane topology predicted by TM-HMM. Column AE includes the complex information where 1 implies that entry is functional as a monomer, 2 as dimer, and so on. Finally, columns AF-AH will be 1 if the protein is a Lon protease target, a lipoprotein, and/or a truncated gene or pseudogene, respectively, 0 otherwise.

Title: Supplementary Data 7

Description: **ProTInSeq signal for *M. pneumoniae* M129 ORFome database.**
First columns (A-AG) are shared with Supplementary Data 6. Following columns include for every sample presenting selective insertion rates in-frame using the following identifiers separated by underscores: marker (BarnB, Cm or Ery), type (control-AC or selection-BD, antibiotic concentration, sample replicate, frame measured, metric. Metrics account for number insertions in-frame (*I*), read count (*R*), linear density from non-coding regions used in the Poisson evaluation (*rNC*), probability measured (*sfNC*) and a binary for prediction (*pred*; 0 - no significant, 1 - significant). Last columns combine the number of samples each annotation has been identified. Notice for barnase library the results need to be interpreted considering it is a negative selection marker inverting the 0 and 1 meaning. This is repeated for all samples from column AH to NS. Last columns (NT-NY) combine the number of samples each annotation has been identified. Notice for barnase library the results need to be interpreted considering it is a negative selection marker inverting the 0 and 1 meaning. These same values by sample can be downloaded at Zenodo under the digital object identifier 10.5281/zenodo.7288780.

Title: Supplementary Data 8

Description: **Summary of the identification method using gene linear densities.**
For different libraries CmB, CmD and EryB, including different concentrations (columns D and E), a ROC curve study is performed retrieving the True Positive Rate (TPR; column G), False Positive Rate (FPR; column H) and Area Under the Curve (AUC; column I). The counts of ORFs estimated with this condition are expressed differentiating between *Ann* (annotated CDS in M. pneumoniae; column J), *New* (putative ORFs; column K), and *Neg* (negative control sequences; column L). The recall (percentage of candidates in each group retrieved) is also included (columns M and N). The ROC values are used to define a sample-specific threshold (*Thr*; column O), required to filter out negative control sequences and keep only those candidates that are significant with no negative control candidates. Following columns include the number of estimated proteins (also separating known SEPs in *M. pneumoniae*; column P-T). Last column includes the length in aa of the shortest ORF identified (column U). Last three rows include the mean values separating the replicas number 4 (corresponding to samples with extra passing selection, colored in purple), and the total unique ORFs identified in each category.

Title: Supplementary Data 9

Description: **Summary for annotated proteins identified with ProTInSeq and other experimental approaches.**
Summary of annotated ORFs and smORFs and the identification methods that report them. Columns A to G include the available information including gene name, start, end and strand in the genome of *M. pneumoniae*, nucleotide and amino acid length, and assigned function in NCBI. Columns H to N are used as binary classification (0 - it is not, 1 - it is in the group) for Lon target, pseudogene, split gene, lipoprotein. transmembrane, signal peptide, and annotated SEP, respectively. Column O and P represent the reference insertion density and essentiality category assigned in the Lluch-Senar, M, *et*

*al.* (2015) study. Columns Q and R include information about how much the subsequences in the gene are repeated. Column S includes the RNA-Seq expression as $log_2$(reads). Columns T to X include the density of insertion in-frame recovered for the selective samples from the study, summarized in column Y as detected (1) or not (0) based on the approach described. These are followed in columns Z to AB for the averaged I - insertions, R - reads, and dens - insertion density when considering those same samples. Columns AC to AH represent the ribosome footprints mapped to the genes by frame and replicate, while columns AI to AK average these same values by replicate and in total. Column AL has the Ribosome Coverage (RCV), calculated as the footprint average normalized by nucleotide length of the annotation, used to define the binary value in column AM. Finally, columns AN to AR include the information extracted from mass spectroscopy (MS), including protein copies per cell, these same values in $log_2$ scale and the bin relative to the distribution, finishing with the protein half life (equal to -10 when no possible to compute) and a binary for detection in MS.

Title: Supplementary Data 10

Description: **Summary of the SEPs identified with ProTInSeq, Ribo-Seq, MS and computational methods.**
Summary of smORFs with significant SEP-coding potential. Columns A to G include the available information including gene name, start, end and strand in the genome of *M. pneumoniae*, nucleotide and amino acid length, and resulting translated sequence. These are followed in columns H to J for the averaged I - insertions, R - reads, and dens - insertion density when considering selective ProTInSeq samples (extracted from Supplementary Data 7). Columns K to S represent the ribosome footprints mapped to the smORFs by frame and replicate, and in average by replicate and in total. Column T has the Ribosome Coverage (RCV), calculated as the footprint average normalized by nucleotide length of the annotation, used to define the binary value in column X. Column U-AA indicates when the SEP is identified by the technique. In order, we include 1 in MS columns when the SEP is detected in one of the 106 mass spectroscopy experiments considered. Column V does the same for SEPs with significant signal by ProTInSeq. Column W has a 1 when the SEP is predicted by the computational approach RanSEPs. Column X for Ribo-Seq based on RCV values. Column Y represents SEPs validated by $C_{13}$ in previous studies and in this study. Column Z indicates when a function has been predicted computationally for a SEP (see Supplementary Data 11). Final column AA represents when the SEP is found by BlastP against the SmProt2 list of SEPs identified by Ribo-Seq in different organisms.

Title: Supplementary Data 11

Description: **Summary of the functions computationally predicted for SEPs.**
Summary of smORFs with significant SEP-coding potential and the computationally predicted functions using different servers and bioinformatic tools. Columns A to F include the available information including gene name, start, end and strand in the genome of *M. pneumoniae*, nucleotide and amino acid length. Column G will present 0 when no information is retrieved by any of the approaches and 1 otherwise. From here, each column header is formatted as a tool:metric provided by the tool. Column H to K presents the results from AMPred, with the last column representing the total count from the three previous values when probability for antimicrobial ≥0.75. Columns L to O present the prediction of protein motifs from PfamScan. Columns P to AI has the complete output from EggNOG. DeepFRI molecular function, GO term and score are included in columns AJ to AL, while columns AM to AO present the PANNZER2 predictions for homologous search in the UniProt database. Column AP to AR present the Phobious output for signal peptide potential predicted by SignalP and transmembrane domains by TMHMM. Final columns AS and AT present the BlastP results as done in Miravet et al. 2019.

Title: Supplementary Data 12

Description: **Estimated number of insertions to obtain similar results in other bacterial species.**

Predicted number of insertions required to achieve similar identification results (highest selection conditions as in *CmB 15*, 75% of genes identified, assuming ~30% of them will be essential as observed in *M. pneumoniae*) in 108 additional bacterial genomes with diverse genome size and number of genes annotated. Column A and B represent a species code and the genome accession considered. These are followed by column C and D with the genome size in total bp and in Kbp. Column E contains the number of annotated genes in NCBI. Column F presents the total number of positions in the genome that are contained in at least one of the genes in the previous column, followed in column G by the number of expected in-frame positions (*i.e.*, first codon positions). The column H (coding ratio) is calculated as the percentage of bp in the genome associated with an annotation. Column I has the total number of ORFs with nucleotide length ≥27 bp. Columns J and K indicate the number of insertions expected with similar conditions for the total genome and for in-frame positions to retrieve comparable results to this study.

Title: Supplementary Data 13

Description: **Ribosome footprints per genome base.**

Ribosome counts per base-pair (column A) representing the number of times a ribosome is found binding an RNA in an exact genome position (discriminating by strand minus and plus) consisting of two biological replicates (RP4 and RP5) from the datasets published in ArrayExpress under the identifier E-MTAB-11935. Processed results are found in Supplementary Data 14.

Title: Supplementary Data 14

Description: **Ribosome profiling metrics for *M. pneumoniae* ORFome.**

For each locus id (column A) different metrics from the ribosome footprints are extracted. Column B and C highlight the type of annotation and alternative name when a locus identifier corresponds to an annotated gene. Columns represent the ribosome footprints mapped to the genes by frame and replicate, while columns D to L average these same values by replicate and in total. Column M has the Ribosome Coverage (RCV), calculated as the footprint average normalized by nucleotide length of the annotation evaluated.
